# Beyond behaviour as individual choice: A call to expand understandings around social science in health research

**DOI:** 10.12688/wellcomeopenres.17149.1

**Published:** 2021-08-23

**Authors:** N Nakkeeran, Emma Sacks, Prashanth N Srinivas, Anika Juneja, Rakhal Gaitonde, Surekha Garimella, Stephanie M Topp

**Affiliations:** 1 School of Global Affairs, Dr. BR Ambedkar University, Delhi, 110006, India; 2 Department of International Health, Johns Hopkins School of Public Health, Baltimore, Maryland, 21218, USA; 3 Health equity cluster, Institute of Public Health, Bangalore, Karnataka, 560070, India; 4 Achutha Menon Centre for Health Science Studies, Sree Chitra Tirunal Institute of Medical Sciences and Technology, Thiruvananthapuram, Kerala, 695011, India; 5 George Institute for Global Health, New Delhi, 110025, India; 6 College of Public Health, Medical and Veterinary Sciences, James Cook University, Townswille, Queensland, 4811, Australia

**Keywords:** behavioural sciences, social sciences, health behaviour, behaviour change, social hierarchies, social conditions

## Abstract

The focus of behavioural sciences in shaping behaviour of individuals and populations is well documented. Research and practice insights from behavioural sciences improve our understanding of how people make choices that in turn determine their health, and in turn the health of the population. However, we argue that an isolated focus on behaviour - which is one link in a chain from macro to the micro interventions - is not in sync with the public health approach which per force includes a multi-level interest. The exclusive focus on behaviour manipulation then becomes a temporary solution at best and facilitator of reproduction of harmful structures at worst. Several researchers and policymakers have begun integrating insights from behavioural economics and related disciplines that explain individual choice, for example, by the establishment of Behavioural Insight Teams, or nudge units to inform the design and implementation of public health programs. In order to comprehensively improve public health, we discuss the limitations of an exclusive focus on behaviour change for public health advancement and call for an explicit integration of broader structural and population-level contexts, processes and factors that shape the lives of individuals and groups, health systems and differential health outcomes.

## Disclaimer

The views expressed in this article are those of the author(s). Publication in Wellcome Open Research does not imply endorsement by Wellcome.

## Introduction

Social sciences have made critical contributions to our understanding of public health as a discipline, in interrogating relationships between the state, institutions, citizens and health, delineating the impact of social hierarchies on health, highlighting the role of governance, policies and power in the preventive, promotive and curative aspects of health, and providing insight in the design, implementation and evaluation of public health interventions. However, the social sciences are often reduced to individual behavioural sciences, which is only one aspect of the broader set of tools, skills, insights, and values that can be brought to improve population health. We, as social scientists, and public health researchers, are particularly concerned about a stream of behavioural studies that is presently dominating the health and public policy field under the *behavioural economics* label. By focusing on the individual level incentive architecture and the cognitive biases documented over the decades and framing these as the sole or pre-dominant level of intervention, these approaches could distract from a more holistic conceptualization of health and its social and societal determinants, as well as the more recently articulated concept of *social determination* ^1, 2^. A focus on the behavioural aspects could become a “quick fix” leaving the larger structural issues unaddressed, addressing the proximal causes of the disease but being blind to the causes of the causes ^3^.

For instance, there was a recent call for papers from the *Bulletin of the World Health Organisation* for a special issue ^4^ on “ *Behavioural and social sciences for better health”*, emphasising contributions from low- and middle income countries. Though admirable for its objective, this call limited itself to the efforts of behavioural sciences almost to the exclusion of other social sciences and used the individual as the only site of intervention. Given the World Health Organization (WHO)'s stated desire to “scale up the use of behavioural and social sciences in public health”, the use of behavioural science to examine factors beyond the control of individuals and the contributions and utilities of other social sciences should be recognized. Academic articles and special issues are one of the ways in which the public health research community shapes its evidence-base and research agendas. Hence, it is crucial that the conceptualisation of the social determination of health is more comprehensive, and that the full complement of social science methodologies and insights are represented, to avoid only including methodologically or socially convenient evidence.

A sole focus on behaviour-shaping (individually or collectively) ignores behavioural and social science contributions that illuminate how structural factors and social hierarchies constitute lives of individuals and communities within which behaviours derive their meaning and relevance. Behavioural health is an essential component of social science, in both research and practice, and many public health programs incorporate behavioural interventions. But public health scholars and professionals have examined many drivers of individual, community, and institutional behaviour, and have shown that interventions must work simultaneously and across many levels in order to achieve desired health outcomes. An exclusive focus on behavioural health with the individual as the sole unit of analysis is flawed and untenable for many reasons. With a number of countries including low- and middle-income countries like India establishing Behavioural Insight Teams (also called *nudge units)* and a number of programs like the Total Sanitation Campaign claiming to rely on insights from behavioural economics in the design and implementation, it is urgent to reflect on these approaches from a more critical perspective including not only the insights from the social sciences but also from larger public health ethics.

We discuss the limitations of an exclusive focus on behaviour change for public health advancement and call for an explicit integration of broader structural and population-level contexts, processes, and factors that shape lives of individuals and groups, health systems and differential health outcomes.

## Conceptualising behaviour

An approach that focuses exclusively or even predominantly on individual behavioural drivers of health considers the individual as the main site of analysis, disregarding existing knowledge of social hierarchies and power relations, and the resulting social oppressions and conflicts. The unfair accumulation of markers of poor health among historically marginalised communities has been demonstrated through many global, national, and sub-national analyses ^5^.

This individual-centred view conceptualises behaviour either as an individual’s unreflective response to situations or as an individual’s choice albeit conditioned by cognitive biases. The former renders aspects like agency and conscious social action invisible, and the latter focuses on 'options' as if they are equally available irrespective of individuals' social locations. Concepts like “collective behaviour” and “steering” seemingly provide an escape from this dichotomy. However, such a restrictive behavioural approach often gets trapped in a language of either victim-blaming or paternalism. Focusing on behaviour as the only effective location to affect change fails to appreciate that most public health problems are interconnected to and nested within layers of health system challenges stemming from social and political contexts.

A behaviourally-focused approach which works only at the individual level implies a presumption in the minds of policymakers and program designers that they know what behaviours are in someone else's best interest ^6^. In a socially differentiated society, it may include additional presumptions on whose behaviours are of interest, manifesting as paternalistic or colonial. At a global level, conceptualising public health phenomena in ways that acknowledge and integrate an equity-focused and decolonised worldview urgently requires recognition of existing social hierarchies and power structures ^7^.

## Recognising social hierarchies and social conditions

An exclusive behavioural focus, not complemented by a social understanding of health, may treat behaviour as an ahistorical and apolitical entity disembodied from social processes. It may fail to adequately problematize the tension between individual and society in conceptualising behaviour. Unravelling the complex contexts and processes through which behaviours get shaped, reified, and differentially valued in society as well as identified and prioritised as relevant ones within public health programming, demands recognition of social hierarchies and power dynamics that operate along these intersecting hierarchies mediated through both distributional and recognition based mechanisms ^8^.

For instance, the problem of open-defecation is addressed as if it were a behaviour that is rooted in individual choice or a cultural practice of a particular social group without adequately provisioning for piped water supply and improved sanitation. This involves the belief that open-defecation can be addressed without a prohibition of manual scavenging or provision of necessary infrastructure. As Link and Phelan emphasise, recognising the link between individual choices in behaviour and “social conditions” is unavoidable and such “fundamental social causes” cannot be addressed by merely readjusting the individually-based mechanisms but only by affecting changes in social conditions ^9^. Methodologically, there has been a stream of behaviour studies that have depended largely on the randomised control trial; while necessary for many scientific questions, a purely experimental approach to such phenomenon as behaviour (even accepting the role of individual cognitive biases) does not do justice to the present understanding of health in all its complexity, and social determination.

Individual behaviours thus should best be seen as emergent manifestation of underlying social and historical processes that shape inequitable access to resources, power and prestige, and differential impacts of market, environment, and policies on people's daily and inter-generational lives ^10^. Comprehensively examining behaviour necessarily includes examining the behaviour and context of institutions and environments. Institutions themselves can be rich sites of analysis, uncovering how scientific evidence translate into policies, and how those policies may be enforced, circumvented, or manifested. Thus, critical social science which examines lived experience, societal and structural factors, and policy environments is necessary to shed light on such complexities.

## Methodological convenience

It is often more difficult to measure and address structural and environmental factors ^11^, rather than individual level factors. Decision-making processes may not be transparent, and power dynamics may be intentionally or unintentionally obtuse. Tracking change longitudinally across many more stakeholders increases the scope and intricacy of measurement efforts.

There is a relative lack of evidence of population-level prevention strategies, which could address upstream social and environmental factors within which health-damaging behavioural choices are made. Such research faces political and cultural obstacles that restrict researchers who are unwilling or unable to produce evidence ^12^ as well as methodological challenges in “studying the complex relationship between the context of health and health outcomes” ^12^. Accounting for politics and social hierarchies, to use Szreter’s words, “makes for a devilishly complicated story" ^13^.

Methodological convenience privileges a reductionist understanding of behavioural and social aspects of health. It is crucial to recognise the multitude of methodological approaches used by behavioural and other social scientists alike, such as ethnography, critical theory, and political economy in fields such as implementation science, anthropology, and policy analysis that lend themselves to exploring, understanding, and conceptualising public health problems as complex social problems. Being inclusive of the many facets of the social sciences will help foster theoretical pluralism, multiple perspectives, and sensitive health systems that engender, promote, and sustain good health.

## Conclusion

Individual behaviour science makes critical contributions towards improved health outcomes but assuming this to be happening in autonomous spaces independent of socio-political contexts and competing interests diminishes its relevance. A call to scale up the application of behavioural and social sciences should promote and utilize approaches with a wider recognition of the scope of behavioural science, and the use of methods which recognize social complexity and address change at the structural and environmental levels. Hence, there is a need for a call that has a more inclusive and transformative view of health that embraces more expansive social sciences.

## Data availability

No data are associated with this article.
