# Supplementary material for: Beyond behaviour as individual choice: A call to expand understandings around social science in health research
Source: Wellcome Open Res. 2021 Aug 23;6:212. [Version 1] doi: 10.12688/wellcomeopenres.17149.1 (PMC8453310; doi:10.12688/wellcomeopenres.17149.1)
Supplement: Peer review history

# Peer review: Reviewer response for version 1

**DOI:** 10.21956/wellcomeopenres.18942.r45590

**Published:** 2021-09-20

**Authors:** Nanda Kishore Kannuri

**Affiliations:** 1 Department of Social and Behavioral Sciences, Indian Institute of Public Health Hyderabad, Public Health Foundation of India, Hyderabad, India

The open letter highlights an important paradigm shaping developmental interventions including public health interventions across the world. The authors make their point that individual behaviour sciences, particularly behavioural economics, are gaining ground at the cost of broader and more holistic public health interventions based on theoretical and methodological underpinnings from social sciences.

 It is a very timely piece that critically analyses a greater policy push to modify individual behaviours and guide them towards a ‘desirable behaviour’ through mainstreaming behavioural economics perspectives.

 I have a few comments on the structure of the article in general and a few suggestions on the content that may help readers to situate the article in a broader debate around public health research.

1. The structure could have been more taut, avoiding a few repetitions in presenting the argument.
2. International readers could benefit if a few more India specific examples on recent nudge based developmental/public health interventions are included.
3. A reference to how public health discipline is largely grounded in a positivist ontology that is defining health research in general.
4. Including a point about the epistemological differences within the social sciences that shape the nature of evidence in public health research and interventions would help the reader to understand the hierarchy of knowledge within the public health discipline.
5. The argument could have been benefited if the article included some critical reflection on: 
  1. The global health push – neoliberal forms of interventions under the rubric of global public health- withdrawal of the state from providing health care, lack of investment in health care infrastructure, individualised public health. 
  2. The role of WHO in shaping the discourse around nature of evidence in public health - while WHO’s commission on social determinants of health put forward a compelling case in 2008 for a public policy that should influence social and environmental factors that shape health inequities, eventually it seems that growing globalisation and neoliberalism, is leading to a renewed shift in WHO towards individual behaviour change model.
6. The reader will also benefit if the authors include a few concrete suggestions in outlining ways forward in their conclusion.

Does the article adequately reference differing views and opinions?

Yes

Are all factual statements correct, and are statements and arguments made adequately supported by citations?

Yes

Is the Open Letter written in accessible language?

Yes

Where applicable, are recommendations and next steps explained clearly for others to follow?

Yes

Is the rationale for the Open Letter provided in sufficient detail?

Yes

Reviewer Expertise:

Medical Anthropology, Social determinants of health, Health Systems, Community based Participatory Appraoches

I confirm that I have read this submission and believe that I have an appropriate level of expertise to confirm that it is of an acceptable scientific standard.

# Peer review: Reviewer response for version 1

**DOI:** 10.21956/wellcomeopenres.18942.r45609

**Published:** 2021-09-14

**Authors:** Marta Schaaf

**Affiliations:** 1 Independent Consultant, New York, NY, USA

This open letter argues that research that centers individual behavior limits our understandings, and thus policy responses, to public health concerns. The letter introduces the topic and then describes different ways that a focus on individual behavior undermines comprehensive understanding. The points are well supported with detail.

 Overall, I found the letter to be clear and making an important argument. My feedback relates to the clarity of the argument and the writing, and can be addressed with minimal effort. Most of my comments relate to the introduction. 

 First, in the abstract, the authors might consider changing the phrase "multi-level interest" to "multi-level framing," which I think more directly communicates the point you are making. Second, the paper refers to the field of behavioural economics, which is being used to inform programs that address both health care workers and community members. However, your article appears to be concerned only with community members. It might be helpful to make this point up front - that you are not writing about programs that try to address the behaviours of HCWs. 

 Third, the introduction mentions "cognitive biases documented over the decades..." I was able to guess what you meant, but I am familiar with and receptive to your argument. It might be helpful to provide some slight clarification (I know you get into it later) about what is meant by the statement about cognitive biases in the introduction.

 Fourth, the term "social determination" is not widely understood, and it might be read by some to be the same as "social determinants." Therefore, I think it would be quite helpful to define this term the first time it is used.

 It might be helpful to your readers (and transparent) to say more about why you feel the WHO Bulletin call for papers was focused on individual level determinants. The letter state "this call limited itself to the efforts of behavioural sciences almost to the exclusion of other social sciences...." You might quote from the call, or, otherwise explain how the call was limited to behavioural sciences almost to the exclusion of other sciences. What justifies your statement?

 The second sentence of the second paragraph is also not clear to me. "Given the WHOs stated desire to 'scale up the use of behavioural and social sciences in public health,' the use of behavioural science to examine the factors beyond the control of individuals and the contributions and utilities of other social sciences should be recognized." Do you mean that the WHO itself realizes the importance of going beyond behavioural science and so their call should reflect this? I read the sentence a few times and did not understand.

 The paper argues that behavioural evidence is "socially convenient." Again, as someone who is primed to receive your argument, I understand what you mean. However, it might be helpful to less "in the know" readers to have a few more words or a description of why this approach is "socially convenient."

 Sometimes it seems as if you are making your argument in very unambiguous terms. For example, the letter states that the Total Sanitation Campaign "claims to rely on insights from behavioural economics." The word "claim" seems gratuitous, as if you are impugning the program designers for saying that it is informed by behavioural economics but it is not actually. Also, do you mean to imply that programs should never be "informed" by behavioural economics? To me, the word "informed" implies that the program designers used insights from behavioural economics; it does not mean that they did not use any other theories or insights. If they only used behavioural economics, then you might state that explicitly.

 The term "manual scavenging" is used in the section on social hierarchies. I don't think that this term is widely understood outside of South Asia.

Does the article adequately reference differing views and opinions?

Yes

Are all factual statements correct, and are statements and arguments made adequately supported by citations?

Yes

Is the Open Letter written in accessible language?

Partly

Where applicable, are recommendations and next steps explained clearly for others to follow?

Yes

Is the rationale for the Open Letter provided in sufficient detail?

Yes

Reviewer Expertise:

Health systems, sexual and reproductive health and rights, human rights, social determinants of health

I confirm that I have read this submission and believe that I have an appropriate level of expertise to confirm that it is of an acceptable scientific standard.
